# Book Reviews

**Published:** 1897-05

**Authors:** 


### BOOK REVIEWS.

LEHRBUCH DER VERGLEICHENDEN PATHOLOGIE UND THERAPIE DES MENSCHEN UND DER HAUSTIERE, FÜR THIERARSTE, ARZTE, UND STUDIRENDE. von DR. GEORG SCHNEIDEMÜHL, Privat Docent der Thiermedizin au der Universitat, Kiel.

TEXT-BOOK OF THE COMPARATIVE PATHOLOGY AND THERAPEUTICS OF MAN AND THE DOMESTIC ANIMALS, FOR VETERINARIANS, PHYSICIANS, AND STUDENTS. By DR. GEORGE SCHNEIDEMÜHL, Instructor of Veterinary Medicine at the University of Kiel. Vol. i., 1895. Vol. ii., 1896. Published by Wilhelm Engelmann, Leipsic, Germany. Price, vol. i., 5 marks ($1.25). Vol. ii., 6 marks ($1.50). (Vols. iii. and iv. to follow.)

THIS book is intended to be a manual for teachers, investigators, and practitioners, from which they can gain all necessary information in regard to the many questions of veterinary science which arise in investigation and practice. By it, it is hoped, the student of human medicine and veterinary medicine may early learn to direct his attention to the most important points of general medicine, and so from the start render the understanding of many questions much easier. But, above all, its purpose is to introduce the veterinarian and the student of veterinary medicine to the study of comparative pathology and therapeutics, and to give in concise form not only a general survey of the subject, but at the same time sufficient detail for practical purposes with reference to standard authorities and hints as to the field of investigation still unoccupied.

In an admirably clear and concise introduction of nine pages the author gives a history of the study of comparative pathology from Hippocrates to Virchow, with the general results so far obtained. Two pages are devoted to the importance of experimental pathology, with many hints as to the methods of procedure and the opportunities which are offered the veterinarian for investigation at present. The general differences in the symptoms and course of diseases in man and in animals are next pointed out, and then general therapeutics is treated, bringing the subject down to the latest discoveries, such as preventive inoculation, etc. The introduction concludes with a survey of the efforts which are being made at present in various countries for further investigation of the subject. Germany, France, England, and the smaller Continental countries are all considered in dealing with the subject.

Vol. I. (200 pages) treats of the infectious diseases of man and the domestic animals.

Vol. II. (240 pages) treats of poisons; diseases of man and animals caused by parasites; constitutional diseases; skin diseaess.

As an example of the method of the author we give a short account of his treatment of glanders, the second disease treated of in Vol. I. Eight pages are devoted to the subject. At the start the scientific name is given; then follow the names applied to the disease in Germany, France, Italy, and England.

Half a page is devoted to the history of the disease; when and by whom it was first noticed; the names of those who have investigated it; how views have changed in regard to it; the latest discoveries, etc. The list of names extends from Aristotle to Virchow, and includes both those who have studied the disease in man and those who have studied it in animals.

Two paragraphs are devoted to the method of spreading and the appearance of the disease; valuable statistics are given and the animals mentioned which enjoy immunity against the disease.

A paragraph is given to the bacterium; its description and conditions of propagation. Another paragraph to its effect upon the animals, the organs affected, and the pathological changes. Lastly, the tests for the bacilli.

Two pages treat of glanders in man under the following heads: Causes, symptoms, and course of the disease; the anatomical conditions after death; diagnosis, prognosis, and therapeutics.

Five pages are devoted to glanders in animals. Causes in the horse, anatomical conditions of the organs affected, symptoms and course of the disease; diagnosis, differential diagnosis in regard to the various phases the disease may take; prognosis and therapeutics.

A half-page is devoted to an account of veterinary police regulations in the various European countries, the attitude of the sanitary police in regard to meat from affected animals, and finally the laws that have been enacted in regard to it in the various European countries.

A good index accompanies the volumes. The text and paper are all that can be desired. The author has drawn on all the standard authorities for the various diseases.

The work appears to have been prepared with the thoroughness and conscientiousness characteristic of the Germans. It is admirable for conciseness and completeness, and we believe that as a manual it admirably fulfils its aim as a help to both the practitioner and the investigator.

One of the best points is that all the very latest statistics, results, and discoveries are contained in it.

---

THE ENCYCLOPÆDIC DICTIONARY. Syndicate Publishing Co., Philadelphia, 1897.

A WORK in four volumes, complete in covering the entire English vocabulary, with those of recent acceptance in the newer fields of scientific investigation, made more valuable by many suggestive illustrations and full-page reproductions, in colors and black, of the greatest interest in elucidating the field covered. We have looked for many of the more common terms, some of the obscure ones used in veterinary science, and found in each one a clear, concise definition, in many instances illustrations, to better afford a clearer conception. With this work in their libraries, veterinarians will be well equipped, will find a great time-saver and a help in reading, an assistant in writing, and a support in better expression of inestimable value. The fourth volume contains a series of tables, of popular terms and expressions, colloquial names, and many other pages of abbreviations and matters of interest, value, and importance to the busy everyday practitioner.

